# Problem Drinking Behaviors: Differential Effects of Stress and School Type on College Students

**DOI:** 10.4236/ojpm.2014.44027

**Published:** 2014-04-02

**Authors:** Alvin Tran, Eric J. Nehl, Jessica Sales, Carla J. Berg

**Affiliations:** Department of Behavioral Sciences and Health Education, Rollins School of Public Health, Emory University, Atlanta, USA

**Keywords:** Problem Drinking Behaviors, College Students, Binge Drinking, Alcohol Use

## Abstract

Given that alcohol use is highly prevalent at US colleges, we explored factors related to problem drinking behaviors (PDB; binge drinking, driving after drinking, sexual intercourse after drinking) among 4098 Black and White students from two- and four-year colleges who completed an online survey. We found an interaction between race and sex such that, among Whites, females had less PDB than males (B = 0.09, CI: 0.05; 0.40, p = 0.01). An interaction between race and school type also existed, such that White students from four-year schools had greater PDB (B = 0.11, CI: 0.20; 0.54, p < 0.001). An interaction between race and stress suggested that Black students were more negatively affected by stress in terms of PBD (B = 0.12, CI: 0.01; 0.07, p = 0.01).

## 1. Introduction

For US college students, alcohol use is highly prevalent [1], and drinking to excess is recognized as a national problem [2]. College students are more likely to consume alcohol and drink more heavily compared to young adults not attending college [3]. While most previous research studying college health programs have focused on traditional four-year colleges [4], little attention has been paid to community college students [5], whose enrollment has experienced a five-fold increase in the past 40 years compared to a doubling of enrollment at four-year colleges [6].

Two commonly reported risk behavior areas coinciding with alcohol consumption and binge drinking include sexual health risk behaviors [7] and driving after alcohol consumption [8]. Frequent and/or heavy alcohol consumption is associated with an increased risk of unprotected sex [9], increased numbers of sexual partners, increased risk of pregnancy, and increased rates of sexually transmitted infection [10]. Moreover, 2.8 million college students report driving under the influence of alcohol, with estimates of alcohol-related traffic deaths among college students ranging from 14.1 to 15.2 deaths per 100,000 [8].

Problem Behavior Theory suggests that problem behaviors, defined as socially problematic, concerning, or undesirable behaviors usually with negative consequences [11], typically result from: 1) the perceived-environment system (e.g., college setting, culture as influenced by race/ethnicity); 2) the personality system (e.g., stress, depression, life satisfaction); and 3) the behavior system. Informed by this framework, the current study aims to examine factors within these three dimensions that impact problem drinking behavior (PDB; *i.e.*, binge drinking, driving after drinking, having sexual intercourse after significant alcohol consumption) among Black and White students attending two- and four-year colleges.

## 2. Methods

### 2.1. Participants

In Fall, 2010, 24,055 students at six Southeast colleges were recruited to complete an online survey. The survey consisted of 230 questions that assessed for a variety of health topic areas, which took approximately 20 – 25 minutes to complete. Students received an e-mail containing a link to the consent form with the alternative of option out. Those who gave consent were directed to the survey; 4849 (20.1%) completed the survey [12]. This study focused on the 4098 students who had complete data and reported their race as being White or Black. As incentive, students received entry into a drawing for cash prizes. The Emory University Institutional Review Board approved this study, IRB# 00030631.

### 2.2. Instrumentation

#### Sociodemographic Characteristics

Students’ age, sex, race/ethnicity, and type of school attended were assessed. Race/ethnicity was categorized as non-Hispanic White or Black given the focus of the current study (*i.e.*, other race/ethnicities were excluded from the analyses).

#### Problem Drinking Behavior (PDB)

To assess PDBs, three questions were asked: 1) “In the past 30 days, on how many of those days did you drink more than 5 alcoholic drinks on one occasion?”; 2) “Did you drink alcohol or use drugs before you had sexual intercourse the last time?” (response options: yes, no, have not had sex); and 3) “During the past 30 days, how many times did you drive a car or other vehicle when you had been drinking alcohol?” For the second question regarding alcohol or drugs prior to the last sexual episode, we found that 88% of students that reported marijuana use also reported frequent alcohol use in the past 30 days; thus, this limitation in the assessment is assumed to minimally impact research findings. An aggregate PDB score was then created, with a range of 0 to 5. The variable alcohol or drugs prior to last intercourse was maintained as a dichotomous variable, with 0 indicating no use prior to last intercourse and 1 indicating use. Binge drinking was recoded into a sub-score of 0 to 2, with 0 indicating no binge drinking, 1 indicating binge drinking 1 – 2 times in the past 30 days, and 2 indicating binge drinking ≥3 days in the past 30 days. Driving after drinking was recoded into a sub-score of 0 to 2, with 0 indicating no drinking and driving, 1 indicating drinking and driving once in the past 30 days, and 2 indicating drinking and driving 2 days in the past 30 days. A score of 5 indicated engaging in all three PDBs and frequently engaging in binge drinking and driving after drinking (Cronbach’s alpha = 0.58).

#### Psychosocial Factors

To assess depression, we administered the Patient Health Questionnaire (PHQ-2) [13], which is a 2-item depression screening tool, based on DSM-4 diagnostic criteria, assessing frequency of depressed mood and anhedonia over the past two weeks (0 = not at all to 3 = nearly every day). To assess perceived stress, we administered the Perceived Stress Scale-4 item (PSS-4) [14], which assesses the degree to which situations in one’s life are appraised as stressful during the last month (0 = never to 4 = very often). To assess satisfaction with life, we administered the Satisfaction with Life Scale (SWLS) [15], which is a 5-item scale designed to measure global cognitive judgments of satisfaction with one’s life (1 = strongly disagree to 7 = strongly agree). Cronbach’s alpha for the PSS-4 and SWLS in the current study was 0.74 and 0.89, respectively.

### 2.3. Analysis

Bivariate analyses were conducted to examine differences in sociodemographic and psychosocial factors in relation to the three dichotomous PDB, using chi-squared tests for categorical variables and t-tests for continuous variables. Sociodemographic and psychosocial factors associated with the aggregate PDB score were then examined using multivariate regression, forcing the correlates of interest into the model. Interactions between race and other sociodemographic and psychosocial factors were also examined in relation to PDB. Statistical significance was set a p = 0.05 for all tests.

## 3. Results

Table 1. provides participant characteristics and bivariate analyses examining sociodemographic and psychosocial variables in relation to the PBD factors. Among the sample, the average PDB index score was 0.54 (SD = 0.84), with 22.9% reporting binge drinking in the past month (10.5% on ≥3 days), 14.2% using alcohol prior to most recent sexual intercourse, and 16.6% reporting driving after drinking in the past month (7.3% on ≥2 days). In the regression models predicting overall PBD score (Table 2), we examined race and its interaction with other factors in relation to PDB and found interactions between: 1) race and sex on PDB, such that White females had lower PDB index scores than White males but Black students not demonstrating this trend; 2) race and the type of school on PDB, such that Whites attending a four-year school had higher PDB indexes than Whites attending a two-year school with Black students not demonstrating this trend; and 3) race and PSS-4 scores on PBD, such that, despite having lower PDB indexes compared to Whites, Black students were more negatively affected by higher perceived stress in terms of PDB.

## 4. Study Limitations

This study’s limitations include a lack of generalizability and a low response rate (20.1%), which may suggest responder bias. Furthermore, previous research has indicated that, despite lower response rates, internet surveys yield similar statistics regarding health behaviors compared to mail and phone surveys [16].

## 5. Discussion

We aimed to determine the sociodemographic and psychosocial factors impacting problem drinking behaviors among black and white students attending two- and four-year colleges. Our research included students from two-year colleges whose nationwide population has shown a five-fold increase over the past 40 years and have been studied less frequently compared to those from four-year colleges [5] [17]. Thus, our paper contributes novel information about a marginalized student population regarding a critical public health issue.

Our study documented novel correlates of PDB, particularly related to race/ethnicity. We found that White females had lower PDB index scores compared to their male counterparts. Previous studies found similar findings such that males accounted for the majority of binge or heavy drinking [18] and that being male was also a predictor of alcohol-impaired driving among college students [19]. Furthermore, Black students demonstrated lower PDB indexes compared to White students overall, which is in line with prior findings [1].

Results also indicated that White students attending four-year schools had higher PDB index scores compared to Whites students attending two-year schools. This effect, however, was not demonstrated among Black students. This difference may be due to the varying social norms, cultures, and environments among two- and four- year academic institutions. Risk factors for alcohol abuse are known to be related to the presence of residence halls, fraternities and sororities, or intercollegiate athletic programs, which are all relatively absent at two-year colleges [20]. There may also be other contextual factors that contribute to these differences and to differential racial experiences of these contextual characteristics among these types of college campuses, which warrants more comprehensive future examination.

In addition, despite having lower PDB indexes, Black students were more negatively affected by higher perceived stress in terms of their PDB compared to White students. While perceived stress levels have previously been found to impact PDB [21] no other research has documented an interaction between race and perceived stress on PDB.

The novel findings presented in this study indicate that potential strategies to address PDB ought to consider the differences among races in relation to sex, campus environments, and reactions to stress as they impact PDB. Further examination of these interactions is warranted in other college student and young adult samples.

Our results have several important implications for future prevention efforts. In addition to the observed inter- actions, our findings suggest that students who are male, attending four-year colleges, and have significant depressive symptoms are most susceptible to engaging in problem drinking behaviors. Thus, future prevention efforts may consider placing greater emphasis on this subgroup. Additionally, while our results suggest that students attending four year colleges have higher problem drinking behavior indexes, we believe that further research is needed to accurately assess the two-year college population where a knowledge gap still exists. Specifically, perceived social norms have been suggested to play a role in being a contributor to drinking among traditional four-year colleges [22] but has less of an impact among two-year colleges where the student population spends less time on campus venues and are less likely to view themselves as traditional college students [23] Thus, potential strategies to address problem drinking behavior ought to consider the differences in regards to the campus and social environments at two- and four-year schools in order to effectively implement prevention and intervention efforts. In addition, interventions that address the issue of depression among students may also assist in countering problem drinking behavior.

## 6. Conclusion

Our study results suggest there are significant interactions between ethnicity with gender, type of school, and perceived stress on problem drinking behavior. In addition, several sociodemographic and psychosocial variables were found to be significantly associated with each of the three problem drinking behavioral factors and may assist in ultimately guiding future public health research and interventions. Additionally, whereas many previous alcohol-related studies have focused on traditional four-year academic institutions, our study included students from both two- and four-year colleges. Ultimately, these findings highlight the need for race/ethnic- and gender-specific interventions that ought to consider the varying environments among two- and four-year colleges. Interventions including a component that addresses stress may also prove to be an effective strategy.

## Figures and Tables

**Table 1:** Bivariate analyses examining factors associated with problem drinking behaviors.

Variable	Total	Binge Drank		p	Drug/Alcohol Use Prior to Last Sex		p	Drove After Drinking		p
		
	Mean (SD) or N (%)	No Mean (SD) or N (%)	Yes Mean (SD) or N (%)		No Mean (SD) or N (%)	Yes Mean (SD) or N (%)		No Mean (SD) or N (%)	Yes Mean (SD) or N (%)	
*Sociodemographic Variables*										
Age (SD)	23.70 (7.39)	23.93 (7.75)	22.98 (5.50)	<0.001	23.64 (7.33)	24.15 (7.17)	0.13	23.64 (7.33)	24.15 (7.17)	0.73
Sex (%)										
Male	1151 (28.1)	715 (24.1)	361 (41.1)	<0.001	869 (26.3)	207 (37.8)	<0.001	843 (26.3)	233 (36.5)	<0.001
Female	2947 (71.9)	2251 (75.9)	518 (58.9)		2429 (73.7)	341 (62.2)		2365 (73.7)	405 (63.5)	
Race (%)										
White	2193 (53.5)	1416 (47.7)	647 (73.6)	<0.001	1707 (51.8)	357 (65.1)	<0.001	1716 (53.5)	348 (54.5)	0.63
Black	1905 (46.5)	1550 (52.3)	232 (26.4)		1591 (48.2)	191 (34.9)		1492 (46.5)	290 (45.5)	
Type of School (%)										
Four-Year	2445 (59.7)	1712 (57.7)	596 (67.8)	<0.001	1964 (59.6)	345 (63.0)	0.14	1850 (57.7)	459 (71.9)	<0.001
Two-Year	1653 (40.3)	1254 (42.3)	283 (32.2)		1334 (40.4)	203 (37.0)		1358 (42.3)	179 (28.1)	
*Psychosocial Variables*										
PHQ-2 (SD)	1.21 (1.31)	1.16 (1.30)	1.39 (1.33)	<0.001	1.17 (1.30)	1.45 (1.36)	<0.001	1.17 (1.30)	1.45 (1.36)	<0.001
PSS-4 (SD)	6.08 (3.42)	5.97 (3.45)	6.48 (3.28)	<0.001	6.02 (3.43)	6.47 (3.35)	0.01	6.02 (3.43)	6.47 (3.35)	0.02
Satisfaction With Life (SD)	22.30 (7.54)	22.49 (7.62)	21.63 (7.32)	0.01	22.52 (7.52)	20.92 (7.53)	<0.001	22.52 (7.52)	20.92 (7.53)	<0.001

**Table 2:** Multivariate regression model indicating factors associated with problem drinking behavior index.

Variable	B	95% CI	p
Age	0.01	(0.00, 0.01)	0.63
Sex			
Male	Ref	-	<0.001
Female	−0.18	(−0.58, −0.36)	
Race			
White	Ref	-	<0.001
Black	−0.43	(−1.44, −0.61)	
Type of School			
Four-year	Ref	-	<0.001
Two-year	−0.21	(−0.62, −0.41)	
PHQ-2	0.09	(0.03, 0.13)	0.001
PSS-4	−0.06	(−0.04, 0.00)	0.06
Satisfaction With Life	−0.09	(−0.02, −0.01)	0.002
Race × Sex	0.09	(0.05, 0.40)	0.01
Race × Type of School	0.11	(0.20, 0.54)	<0.001
Race × PHQ2	−0.04	(−0.12, 0.03)	0.24
Race × PSS-4	0.12	(0.01, 0.07)	0.01
Race × Satisfaction With Life	0.05	(−0.01, 0.02)	0.41

## References

[1] O’Malley PM, Johnston LD (2002). Epidemiology of Alcohol and Other Drug Use among American College Students. Journal of Studies on Alcohol and Drugs.

[2] Task Force of the National Advisory Council on Alcohol Abuse and Alcoholism (2002). How to Reduce High-Risk College Drinking: Use Proven Strategies, Fill Research Gaps. Final Report of the Panel on Prevention and Treatment.

[3] Grucza RA, Norberg KE, Bierut LJ (2009). Binge Drinking among Youths and Young Adults in the United States: 1979–2006. Journal of the American Academy of Child and Adolescent Psychiatry.

[4] Townsend B, Donaldson J, Wilson T (2005). Marginal or Monumental? Visibility of Community College in Selected Higher-Education Journals. Community College Journal of Research and Practice.

[5] Chiauzzi E, Donovan E, Black R, Cooney E, Buechner A, Wood M (2011). A Survey of 100 Community Colleges on Student Substance Use, Programming, and Collaborations. Journal of American College Health.

[6] National Center for Educational Statistics (2007). Degree Granting Institutions, by Control and Type of Institution: 1963 through 2005.

[7] Pedrelli P, Bitran S, Shyu I, Baer L, Guidi J, Tucker DD, Vitali M, Fava M, Zisook S, Farabaugh AH (2011). Compulsive Alcohol Use and Other High-Risk Behaviors among College Students. American Journal on Addictions.

[8] Hingson RW, Zha W, Weitzman ER (2009). Magnitude of and Trends in Alcohol-Related Mortality and Morbidity among US College Students Ages 18–24, 1998–2005. Journal of Studies on Alcohol and Drugs.

[9] Graves KL (1995). Risky Sexual Behavior and Alcohol Use among Young Adults: Results from a National Survey. American Journal of Health Promotion.

[10] Santelli JS, Brener ND, Lowry R, Bhatt A, Zabin LS (1998). Multiple Sexual Partners among US Adolescents and Young Adults. Family Planning Perspectives.

[11] Jessor R, Jessor S (1977). Problem Behavior and Psychological Development: A Longitudinal Study of Youth.

[12] Berg CJ, Nehl E, Sterling K, Buchanan TS, Narula S, Sutfin E, Ahluwalia JS (2011). The Development and Validation of a Scale Assessing Individual Schemas Used in Classifying a Smoker: Implications for Research and Practice. Nicotine & Tobacco Research.

[13] Kroenke K, Spitzer RL, Williams JB (2003). The Patient Health Questionnaire-2: Validity of a Two-Item Depression Screener. Medical Care.

[14] Cohen S, Kamarck T, Mermelstein R (1983). A Global Measure of Perceived Stress. Journal of Health & Social Behavior.

[15] Diener E, Emmons RA, Larsen RJ, Griffin S (1985). The Satisfaction With Life Scale. Journal of Personality Assessment.

[16] An LC, Hennrikus DJ, Perry CL, Lein EB, Klatt C, Farley DM, Bliss RL, Pallonen UE, Lando HA, Ehlinger JS, Ahluwalia JS (2007). Feasibility of Internet Health Screening to Recruit College Students to an Online Smoking Cessation Intervention. Nicotine & Tobacco Research.

[17] National Center for Educational Statistics, National Center for Educational Statistics (2007). Degree Granting Institutions, by Control and Type of Institution: 1963 through 2005.

[18] Cranford JA, McCabe SE, Boyd CJ (2006). A New Measure of Binge Drinking: Prevalence and Correlates in a Probability Sample of Undergraduates. Alcoholism: Clinical and Experimental Research.

[19] Wechsler H, Davenport A, Dowdall G, Moeykens B, Castillo S (1994). Health and Behavioral Consequences of Binge Drinking in College. A National Survey of Students at 140 Campuses. JAMA.

[20] Ryan B (1998). Alcohol and Other Drugs: Prevention Challenges at Community Colleges.

[21] Rice KG, Van Arsdale AC (2010). Perfectionism, Perceived Stress, Drinking to Cope, and Alcohol-Related Problems among College Students. Journal of Counseling Psychology.

[22] Sheffield FD, Darkes J, Del Boca FK, Goldman MS (2005). Binge Drinking and Alcohol-Related Problems among Community College Students: Implications for Prevention Policy. Journal of American College Health.

[23] Sher KJ, Bartholow BD, Nanda S (2001). Short- and Long-Term Effects of Fraternity and Sorority Membership on Heavy Drinking: A Social Norms Perspective. Psychology Addictive Behaviors.

